# Revisiting patient satisfaction following total knee arthroplasty: a longitudinal observational study

**DOI:** 10.1186/s12891-018-2340-z

**Published:** 2018-11-30

**Authors:** Stirling Bryan, Laurie J. Goldsmith, Jennifer C. Davis, Samar Hejazi, Valerie MacDonald, Patrick McAllister, Ellen Randall, Nitya Suryaprakash, Amery D. Wu, Richard Sawatzky

**Affiliations:** 1 Centre for Clinical Epidemiology & Evaluation, Vancouver Coastal Health Research Institute, West 10th Avenue, Vancouver, BC V5Z 1M9 Canada; 2 School of Population & Public Health, University of British Columbia, 2206 E Mall, Vancouver, BC V6T 1Z3 Canada; 3 Faculty of Health Sciences, Simon Fraser University, 8888 University Drive, Burnaby, BC V5A 1S6 Canada; 4 Faculty of Management, University of British Columbia – Okanagan Campus, EME 4145 3333 University Way, Kelowna, BC V1V 1V7 Canada; 5 Department of Evaluation & Research Services, Fraser Health Authority, Suite 400, Central City Tower, 13450 102 Avenue, Surrey, BC V3T 0H1 Canada; 6 Burnaby Hospital & Surgical Network, Fraser Health Authority, 3935 Kincaid St, Burnaby, BC V5G 2X6 Canada; 7 Rebalance MD, 3551 Blanshard St, Victoria, BC V8Z 0B9 Canada; 8 Educational and Counselling Psychology, and Special Education, University of British Columbia, 2125 Main Mall, Vancouver, BC V6T 1Z4 Canada; 9 School of Nursing, Trinity Western University, 7600 Glover Rd, Langley, BC V2Y 1Y1 Canada; 10 Centre for Health Evaluation & Outcomes Sciences, Providence Health Care Research Institute, 588 – 1081 Burrard Street, St. Paul’s Hospital, Vancouver, BC V6Z 1Y6 Canada

**Keywords:** Total knee arthroplasty, Patient satisfaction, Longitudinal observational study, Survey research

## Abstract

**Background:**

Total knee arthroplasty (TKA) is the most common joint replacement surgery in Canada. Earlier Canadian work reported 1 in 5 TKA patients expressing dissatisfaction following surgery. A better understanding of satisfaction could guide program improvement. We investigated patient satisfaction post-TKA in British Columbia (BC).

**Methods:**

A cohort of 515 adult TKA patients was recruited from across BC. Survey data were collected preoperatively and at 6 and 12 months, supplemented by administrative health data. The primary outcome measure was patient satisfaction with outcomes. Potential satisfaction drivers included demographics, patient-reported health, quality of life, social support, comorbidities, and insurance status. Multivariable growth modeling was used to predict satisfaction at 6 months and change in satisfaction (6 to 12 months).

**Results:**

We found dissatisfaction rates (“very dissatisfied”, “dissatisfied” or “neutral”) of 15% (6 months) and 16% (12 months). Across all health measures, improvements were seen post-surgery. The multivariable model suggests satisfaction at 6 months is predicted by: pre-operative pain, mental health and physical health (odds ratios (ORs) 2.65, 3.25 and 3.16), and change in pain level, baseline to 6 months (OR 2.31). Also, improvements in pain, mental health and physical health from 6 to 12 months predicted improvements in satisfaction (ORs 1.24, 1.30 and 1.55).

**Conclusions:**

TKA is an effective intervention for many patients and most report high levels of satisfaction. However, if the TKA does not deliver improvements in pain and physical health, we see a less satisfied patient. In addition, dissatisfied TKA patients typically see limited improvements in mental health.

## Background

Total knee arthroplasty (TKA) is the most commonly performed joint replacement surgery in Canada where there were over 67,000 TKAs in 2016/17 [1]. TKA is typically performed on people with osteoarthritis, the prevalence of which increases with age, and so demand for TKA can be expected to continue to rise given population demographic trends [2–4]. Through 2015/16, Canada saw a 5-year increase in TKA procedures of 16% [1]. A commonly cited and troubling statistic is that approximately 1 in 5 TKA patients express some dissatisfaction with their outcomes following surgery [5, 6]. The concern is magnified when placed in the contemporary context of health system commitments to patient-centred care, with consideration of traditionally ignored outcomes such as patient satisfaction and quality of life [7, 8]. Using a patient-centred lens, a dissatisfied TKA patient has not had a successful surgery, and a rate of 20% dissatisfied patients points to a need for improvement. A better understanding of patient satisfaction drivers could guide program improvement initiatives.

Previous studies have explored the relationship between post-TKA patient satisfaction and various combinations of pre- and post-surgery clinical and patient-reported measures. Factors found to be related to patient dissatisfaction across multiple studies include: knee-related factors (e.g., pain, functioning, stiffness and inflammation), self-rated factors (e.g., physical and mental health status, and quality of life), pre-surgery expectations not met, complications, pain catastrophizing, and patient demographics (e.g., age, gender and employment status) [5, 9–19]. The existing literature typically reports analyses of post-TKA patient satisfaction at a single time, even if repeated measurements (most commonly at 6- and 12-months) were made, and so contributes very little to understanding changes in satisfaction over time [20–23]. For example, are the concerns of patients in the early post-operative period different from those dealing with longer-term dissatisfaction? That requires a longitudinal analysis which simultaneously adjusts for correlations between repeated measures [24].

The longitudinal observational study reported in this paper was designed to understand patient satisfaction with TKA at 6 and 12-months post-surgery, as well as changes in satisfaction over time. The paper reports the quantitative work from a multiphase, longitudinal, mixed methods study investigating patient satisfaction following TKA surgery [25]. Using a patient-centred perspective, we investigated patient satisfaction rates post-TKA in British Columbia (BC), with exploration of the primary drivers of variation in the level of, and change in, patient satisfaction following TKA.

## Methods

### Setting, sample and data collection

A cohort of 515 patients was recruited from six hospital sites spanning all regional health authorities in BC. Consecutive patients (aged 19 years or older) with a primary or secondary diagnosis of osteoarthritis, and scheduled to undergo primary TKA at one of the six sites, were invited to participate during mandatory pre-surgical total joint replacement education sessions. Patients scheduled to undergo revision surgery, bilateral knee replacement, unicompartmental knee replacement, or TKA due to an accident were excluded. The participation rate was 57% (515/808) out of all invited patients. Approval was obtained from research ethics boards of all health authorities and universities in the study.

Much of the study data were collected using a preoperative survey questionnaire (administered up to 3 months before surgery) and two post-operative survey questionnaires (at 6 and 12 months post-surgery). The questionnaires were self-administered in English, with family members or caregivers serving as translators for non-English speaking patients. All non-respondents received reminders. Retention rates were 91% (466/515) and 88% (455/515) at 6 and 12 months, respectively. In addition, health administrative data were obtained from medical records of consenting patients (93%, 479/515).

### Measurements and outcomes

The primary outcome measure was patient satisfaction with the results of their knee surgery, collected at 6 and 12 months. These intervals were chosen to mirror previous work in TKA [5], and endorsed by clinical experts on our team. Participants were asked to respond to a single-item measuring satisfaction with the outcomes of knee surgery based on a 5-point Likert rating (varying from “very satisfied” through “very dissatisfied”), which was previously used in a large Canadian cohort study of knee arthroplasty outcomes [4]. For analysis purposes, the primary satisfaction outcome variable was collapsed into a binary variable with values 0 (“very satisfied” or “satisfied”) and 1 (“very dissatisfied”, “dissatisfied” or “neutral”).

Potential patient-centred drivers of satisfaction explored in our analyses, based on the literature review, included demographic variables, patient-reported health status (SF-12 [26], EQ-5D-5 L [27], WOMAC [28], SLANSS [29]), depression and anxiety (HADS [30]), social support (MOS-SSS [31]), patient expectations, comorbidities (Charlson [32]), health insurance, and global quality of life (Cantril [33]). All selected instruments have been used previously in TKA patients, with strong evidence for their validity [26–33].

In addition to patient-reported measures, administrative data were extracted from in-patient medical records to measure hospital length of stay during the surgical period, complications following surgery, re-admissions and presence of co-morbidity during the inpatient stay. The patient’s postal code information was used to classify rural/urban residence and to measure distances from the patient’s home to the hospital where they had their TKA surgery, and the nearest hospital where TKA is offered.

### Analyses

Multivariable growth modeling was conducted using the M*Plus* software (version 7.4) [34] to predict individual differences in satisfaction at 6 months (intercepts) and change (slopes) in satisfaction from 6 to 12 months [35, 36]. The model was specified as a logistic regression, with satisfaction as a binary variable and random effects for the intercepts and slopes. Accordingly, the results are presented as odds ratios (ORs) pertaining to the intercepts and slopes associated with the independent variables. To minimize collinearity issues resulting from high correlations over time, the time-varying predictor variables were represented as pre-surgery scores, the change from pre-surgery to 6 months post-surgery, and the change from 6 to 12 months post-surgery. All continuous predictor variables were rescaled to vary from 0 to 10 to enhance interpretability and comparisons for the odds ratios. Full information maximum likelihood estimation was applied to accommodate for missing data.

The model was built sequentially where the selection of variables was informed by prior studies, findings from our qualitative analysis [37], and emerging statistical results, including changes in overall model fit and parameter estimates [38, 39]. First, the patient-reported outcome variables (WOMAC subscales, SF-12 mental and physical health component scores, the EQ-5D valuation score) were examined. To avoid multicollinearity, variable selection at this step was guided by identifying a set of variables that were correlated with satisfaction and least correlated with one another. Second, demographic variables (age, sex, health region, marital status, education, ethnicity, and urban versus rural) were entered into the model. The third and final step involved examining other health-related predictors, including neuropathic pain (SLANSS), depression and anxiety (HADS), social support (MOS SSS), comorbidities (Charlson), health insurance, and global quality of life (Cantril). At each of steps 2 and 3, all variables were first entered simultaneously and the models were subsequently trimmed by sequentially removing variables that were not statistically significant predictors (*p* < .05) while monitoring the impact on overall model fit, based on the Bayesian Information Criterion (BIC), and changes in parameter estimates.

## Results

The characteristics of the patient cohort at the time of recruitment into the study are reported in Table 1. The cohort had a mean age of 66 years, was predominantly female (61%), over one-quarter was still working and over half waited longer than 12 months for surgery. Almost one-third of patients had no supplementary health insurance to cover the costs of additional health care services such as privately-financed physiotherapy over and above the standard series of publicly provided physiotherapy.

**Table 1:** Time Invariant Variables at Baseline

Variable	Mean (SD) / Frequency (%)
Age in years (*n* = 515)	66.5 (9.0)
Sex (*n* = 511)	
Male	200 (39)
Female	311 (61)
Marital Status (*n* = 509)	
Married or Common law	353 (69)
Widowed	54 (11)
Separated or Divorced or Single	102 (20)
Working Status (*n* = 510)	
No	375 (74)
Yes	135 (26)
Annual Household Income (*n* = 471)	
< $40 k	168 (36)
$40 k - $60 k	122 (26)
$60 k - $80 k	60 (13)
> $80 k	121 (26)
Education (*n* = 464)	
< High school	72 (16)
High school	144 (31)
College/Technical	136 (29)
University degree undergraduate	112 (24)
Other	37 (8)
Ethnicity (*n* = 505)	
North American	309 (61)
European	134 (27)
South Asian Indian Pakistani Bangladesh	29 (6)
Pacific Asian Chinese Japanese Korean	17 (3)
Other	16 (3)
Supplementary insurance (*n* = 459)	
No	148 (32)
Yes	311 (68)
Expected pain 3 months after surgery (*n* = 502)	
Not at all painful	150 (30)
Slightly painful	238 (47)
Moderately painful	101 (20)
Very painful	13 (3)
Expected limitation in usual activity 3 months after surgery (*n* = 501)	
Not at all limited	124 (25)
Slightly limited	246 (49)
Moderately limited	117 (23)
Very limited	14 (3)
Urban or rural (*n* = 497)	
Urban	445 (86)
Rural	52 (10)

Table 2 reports the results of the primary outcome variable, satisfaction with outcomes (at both 6 and 12 months post-surgery). Our data indicate an overall dissatisfaction rate (“very dissatisfied”, “dissatisfied” or “neutral”) of 15% at 6 months and 16% at 12 months post-surgery. The vast majority of participants (78%) were satisfied or very satisfied with their outcomes at both time points, and 8% indicating some level of dissatisfaction (or neutrality) at both time points.

**Table 2:** Distribution of satisfaction with outcomes at 6 and 12 months

	Satisfaction at 12 months						
Satisfaction at 6 months	Very dissatisfied	Dissatisfied	Neutral	Satisfied	Very satisfied	Missing	Total
Very dissatisfied	2	0	0	1	4	3	10
Dissatisfied	4	3	4	5	1	0	17
Neutral	1	9	11	13	2	7	43
Satisfied	0	8	16	85	37	9	155
Very satisfied	7	0	2	39	176	14	238
Missing	1	0	4	5	11	31	52
Total	15	20	37	148	231	64	515

Self-reported patient health outcomes over time are reported in Table 3. Our data point to major improvements in patient health post-surgery; a finding seen across all outcome instruments. The improvement is most clearly seen from baseline to 6 months, with very little further improvement beyond 6 months. Improvements were seen in quality of life, pain, physical function and mental health.

**Table 3:** Time varying variables

		Mean (SD)		
Instrument	Component	Baseline (n = 515)	6 months (*n* = 463)	12 months (*n* = 451)
EQ-5D	Visual Analogue Scale (0–100; higher score is better)	69.2 (17.7)	78.3 (15)	78.2 (14.5)
EQ-5D utility	Utility score (−0.148 to 1; higher positive score is better)	0.59 (0.21)	0.82 (0.14)	0.82 (0.14)
SF-12	Physical Composite Scale (PCS) Score (0–100; higher score is better)	34.3 (7.5)	43.2 (12.6)	44.9 (9.2)
	Mental Health Composite Scale (MCS) Score (0–100; higher score is better)	51.6 (10.7)	54.0 (8.8)	54.0 (8.7)
WOMAC	Pain (0–20; higher score, more pain)	10.1 (3.6)	3.4 (3.0)	2.9 (3.2)
	Stiffness (0–8; higher score, more stiffness)	4.2 (1.7)	2.1 (1.6)	1.7 (1.5)
	Physical Function (0–68; higher score, more limitations)	33.8 (11.9)	13.2 (11.2)	12.4 (11.5)
HADS	Anxiety (0–21; higher score, more anxiety)	5.6 (3.9)	4.0 (3.4)	4.1 (3.6)
	Depression (0–21; higher score, more depression)	4.8 (3.3)	3.1 (3.2)	3.3 (3.1)
SLANSS	Neuropathic pain (0–24; score > =12, pain of neuropathic origin)	7.0 (6.6)	6.4 (6.9)	5.7 (6.4)

The final multivariable model predictors of satisfaction are shown in Table 4. This represents the best fitting and most parsimonious model predicting satisfaction and change in satisfaction scores. The multilevel multivariable regression results suggest that self-reported pre-operative pain, based on the WOMAC, as well as the difference in pain at 6 months were predictive of satisfaction at 6 months. In addition, pre-operative mental and physical health are both predictive of satisfaction at 6 months (ORs 3.25 and 3.16, respectively). Change in pain from 0 to 6 months was also predictive of satisfaction at 6 months (OR = 2.31). However, changes in mental and physical health from 0 to 6 months were not. Further, a change in any of the three variables (pain, mental health and physical health) from 6 to 12 months was predictive of a change in satisfaction; people experiencing improved pain, mental health or physical health were more likely also to experience improved satisfaction from 6 to 12 months (ORs 1.30, 1.55 and 1.24, respectively).

**Table 4:** Final multivariable model results

Variable	Time	Estimate	SE	*p*	OR^1^ (95%CI)
*Predictors of the intercept*					
SF-MCS	1	0.118	0.034	0.001	3.25 (1.67–6.34)*
	2–1	0.01	0.026	0.686	1.11 (0.66–1.84)
SF-PCS	1	0.115	0.038	0.002	3.16 (1.50–6.65)*
	2–1	0.026	0.036	0.473	1.30 (0.64–2.63)
WOMAC PAIN^2^	1	0.418	0.111	< 0.001	2.65 (1.76–4.01)*
	2–1	0.488	0.105	< 0.001	2.31 (1.49–3.56)*
*Predictors of the slope*					
SF-MCS	3–2	0.026	0.011	0.014	1.30 (1.05–1.61)*
SF-PCS	3–2	0.044	0.016	0.005	1.55 (1.13–2.12)*
WOMAC PAIN^2^	3–2	0.109	0.034	0.001	1.24 (1.09–1.42)*
Correlation intercept and slope		−1.161	2.184	0.595	
slope (mean)		−0.334	0.433	0.441	
intercept (threshold)		2.244	2.384	0.346	
Residual variance: intercept		8.851	9.003	0.326	
Residual variance: slope		0.159	0.417	0.703	

Notes Time 1 is baseline, Time 2 is 6 months, and Time 3 is 12 months. *Log likelihood* − 227.67, *BIC* 548.03, ^*1*^*OR* odds ratio pertaining to a 10% difference in scores of the continuous independent variables comparing the odds of being in a higher satisfaction category relative to a lower satisfaction category. ^2^Reverse-scored, such that higher score indicates less pain

Different combinations of the core patient-reported outcome variables (including the SF-36 component scores, the WOMAC subscales, and EQ-5D) were explored, but none resulted in additional statistically significant parameter estimates and improved model fit (based on the BIC). The addition of individual time-invariant variables (marital status, age, gender, comorbidity, and having additional health insurance) also did not result in any improvement in model fit, regardless of whether the variables were entered individually or in combination with one or more other time-invariant variables. Model fit was also not improved by any of the other patient-reported outcome variables (global quality of life, neuropathic pain, anxiety, depression and social support) when entered into the model as pre-operative scores and their difference scores at 6 and 12 months.

In summary, a key driver of patient satisfaction post-surgery is pain (both pre-surgery and the change in pain levels over time). The other factors associated with patient satisfaction post-surgery are both physical health (over and above pain) and mental health. Many other factors were found not to have an association with patient satisfaction, notably socio-demographic characteristics of patients, patient expectations and level of support.

## Discussion

### Summary of main findings

Our data indicate that TKA is an effective intervention for many recipients, with major gains in health-related quality of life reported by those who receive the procedure. Further, most patients report high levels of satisfaction post-surgery: we found an overall dissatisfaction rate among Canadian TKA patients of approximately 16% at 12 months post-surgery. Although this dissatisfaction rate, in aggregate, remains stable from 6 to 12 months post-surgery, we do see movement over time; some patients indicating increasing dissatisfaction and others moving in the opposite direction. When looking to understand factors associated with patient satisfaction, the longitudinal nature of our data and analyses allow us to tease out the impact of pre-surgery measurements and changes over time. Our results indicate three key satisfaction drivers: pain, physical health and mental health. Pre-surgery levels of all three variables were found to be important predictors of satisfaction at 6 months; and changes in the levels of all three (from 6 to 12 months post-surgery) were strongly associated with changes in satisfaction. In summary, if the TKA procedure has positive impacts on a patient’s pain levels and overall physical health, we are likely to see a satisfied patient. However, and more surprisingly, we also see that satisfied TKA patients are also likely to have seen some improvements in relation to mental health too.

### Comparison with previous work

Our study resembles others in finding knee pain to be a key predictor of post-TKA satisfaction [13, 16, 40, 41]. This common finding holds for both pre-surgical pain and post-surgery pain improvement. Our study also reinforces findings by others that pre-surgical physical and mental health are positively related to post-TKA patient satisfaction [13, 16, 19, 41, 42]. However, given our longitudinal analysis, our study departs from the existing literature in being able to comment on contributors to change in satisfaction rates over time. As far as we know, our principal findings of a relationship between changes in pain, physical health status and mental health status and changes in patient satisfaction have not been shown previously.

### Strengths and weaknesses of our study

One of the main strengths of our research is the longitudinal design and analysis of the research. Ours is not the first study of knee replacement experience to measure satisfaction longitudinally, but it is the first to employ methods that directly incorporate the longitudinal data into the analysis. Another major strength is the retention rates achieved; approximately 90% of the cohort returned surveys at both 6 and 12 months, with very little loss over time. However, we also recognize that many patients invited to participate in this research chose not to do so, the consequence being a challenge to the representativeness of the sample.

The limited ethnic diversity in our sample is a weakness, resulting in part from the fact that we were able only to use English language surveys and study materials. Further work is required to establish the generalizability of our findings to other major ethnic groups in British Columbia and Canada more generally. We selected a measure of patient satisfaction used previously in a Canadian TKA context, in part to facilitate direct comparison with earlier Canadian work [5]. We do, however, acknowledge weaknesses with the satisfaction measure, notably its focus on satisfaction with results only, as opposed to a broader measure of patient experience. Our sample of patients all received care in BC, with recruitment from all regions of the province, reflecting both urban and more rural settings. To facilitate the recruitment process, we targeted pre-surgery education sessions and so we were only able to recruit from hospital sites that offered such education. Finally, other variables not measured in this study may explain some variation in satisfaction. For example, we had data on supplementary health insurance status but not on which patients might have augmented the standard series of publicly-provided physiotherapy with additional privately-financed physiotherapy or on the total number of private physiotherapy sessions received. We would encourage future research on this topic to consider gathering additional clinical data (e.g., clinical indication for surgery such as pain or joint surface destruction; clinical outcomes such as knee alignment; services provided such as number of physiotherapy sessions, and patient out of pocket costs) that might point directly to actions for improvement.

## Conclusions

Our research confirms the importance of pain and functioning post-surgery as key drivers of patient satisfaction. Ongoing monitoring of such patient-reported factors, and intervention in cases where such symptoms persist, is central to patient satisfaction. Another key finding of our work is that a patient’s mental health also heavily influences satisfaction post-TKA. Both the level of self-reported mental health and changes in that level over time predict satisfaction rates. This important finding points to the need for broad clinical review of patients, before and after surgery that encompasses both physical and mental health aspects. Screening for and addressing mental health concerns will likely be a new domain for many surgical orthopedic programs. The priority research implications of this work are two-fold. First, we need to explore the robustness of our results across more ethnically diverse British Columbian and Canadian patient populations. Second, the effectiveness and cost-effectiveness of any new interventions targeting the drivers of dissatisfaction uncovered in our work will need to be tested empirically. For example, a response to our findings might be to consider new interventions to promote mental health and wellness amongst patients receiving TKA but this should, of course, be subjected to evaluation of its costs and benefits before implementation.

## Abbreviations

BC: British Columbia
BIC: Bayesian Information Criterion
CIHI: Canadian Institutes for Health Information
EQ-5D-5 L: Euroqol 5 dimension, 5 level instrument
HADS: Hospital Anxiety and Depression Scale
MOS-SSS: Medical Outcomes Study Social Support Survey
OR: Odds ratio
SF-12: Short-form 12 instrument
SLANSS: Self-completed Leeds Assessment of Neuropathic Symptoms and Signs pain scale
TKA: Total knee arthroplasty
WOMAC: Western Ontario and McMaster Universities Osteoarthritis Index
